# Preliminary Evidence for a Hormetic Effect on DNA Nucleotide Excision Repair in Veterans with Gulf War Illness

**DOI:** 10.1093/milmed/usz177

**Published:** 2019-07-23

**Authors:** Jean J Latimer, Abdullah Alhamed, Stefanie Sveiven, Ali Almutairy, Nancy G Klimas, Maria Abreu, Kimberly Sullivan, Stephen G Grant

**Affiliations:** 1 Department of Pharmaceutical Sciences, Nova Southeastern University, 3200 South University Drive, Fort Lauderdale, FL 33328; 2 South University Drive, AutoNation Institute for Breast Cancer Research, 3321, Fort Lauderdale, FL 33328; 3 Department of Clinical Immunology, Nova Southeastern University, 3200 South University Drive, Fort Lauderdale, FL 33328; 4 Department of Medicine, Miami VA Healthcare System, 1201 NW 16th St, Miami, FL 33313; 5 Department of Environmental Health, Boston University School of Public Health, 715 Albany St, Boston, MA 02118; 6 Department of Public Health, Nova Southeastern University, 3200 South University Drive, Fort Lauderdale, FL 33328

**Keywords:** Gulf War Illness, DNA repair, NER, gene expression, exposure response

## Abstract

**Introduction:**

Veterans of the 1991 Gulf War were potentially exposed to a mixture of stress, chemicals and radiation that may have contributed to the persistent symptoms of Gulf War Illness (GWI). The genotoxic effects of some of these exposures are mediated by the DNA nucleotide excision repair (NER) pathway. We hypothesized that individuals with relatively low DNA repair capacity would suffer greater damage from cumulative genotoxic exposures, some of which would persist, causing ongoing problems.

**Materials and Methods:**

Blood samples were obtained from symptomatic Gulf War veterans and age-matched controls. The unscheduled DNA synthesis assay, a functional measurement of NER capacity, was performed on cultured lymphocytes, and lymphocyte mRNA was extracted and analyzed by sequencing.

**Results:**

Despite our hypothesis that GWI would be associated with DNA repair deficiency, NER capacity in lymphocytes from affected GWI veterans actually exhibited a significantly elevated level of DNA repair (*p* = 0.016). Both total gene expression and NER gene expression successfully differentiated individuals with GWI from unaffected controls. The observed functional increase in DNA repair capacity was accompanied by an overexpression of genes in the NER pathway, as determined by RNA sequencing analysis.

**Conclusion:**

We suggest that the observed elevations in DNA repair capacity and NER gene expression are indicative of a “hormetic,” i.e., induced or adaptive protective response to battlefield exposures. Normally such effects are short-term, but in these individuals this response has resulted in a long-term metabolic shift that may also be responsible for the persistent symptoms of GWI.

## INTRODUCTION

Veterans who served in the 1991 Gulf War report debilitating health symptoms 2–3× more frequently than military personnel who were not deployed to the Gulf.^1^ These symptoms are multi-system and non-specific, involving fatigue, headache, memory problems, sleep disorders, respiratory problems and musculoskeletal pain.^2,3^ Gulf War illness (GWI) is a life-altering disease presumably caused by multiple cumulative exposures.^4–7^ Since some, but not all personnel manifest this disease, there may be an additional genetic component that increases vulnerability to such exposures.^8–10^

A major component of the exposures documented in this population was smoke from oil well fires set by the retreating Iraqis.^11–13^ The activated products of organic combustion created in such fires are known to form “bulky” adducts in DNA,^14^ and such damage lesions are substrates for the DNA nucleotide excision repair (NER) pathway.^15,16^ We therefore analyzed NER capacity and gene expression in veterans with GWI to determine whether they had reduced DNA repair and were therefore more susceptible to the genotoxic effects of Gulf War exposures.

## METHODS

### Subjects

Gulf War veterans were recruited as part of ongoing studies.^5^ Seven blood samples were obtained, from six veterans diagnosed with GWI by the Kansas criteria and one asymptomatic veteran who had also been deployed in the Gulf War (all men).^17^ Three local controls of appropriate age (range 39–60) were also recruited, sampled and analyzed (two male and one female). All samples were collected under MVAMC IRB Protocol 4987.78 “Brain-immune interactions as the basis of Gulf war illness consortium (GWIC)”. For functional analysis of NER, eight samples of appropriate age (range 27–59) were utilized as historical controls (including six women and two men).^18,19^

### Unscheduled DNA Synthesis Assay

Functional NER capacity was analyzed in cultured blood lymphocytes using the unscheduled DNA synthesis (UDS) assay as previously described.^20^ From each blood sample, the buffy coat was isolated by centrifugation. For culture of isolated lymphocytes, a basement membrane layer (Cultrex Basement Membrane Extract, Trevigen) was applied to two 2-chamber glass slides (Nunc Lab-Tek Chamber Slide System, Thermo Scientific). The buffy coat was brought to a total volume of 4mLs in RPMI + 10% FBS + 3× antibiotic-antimycotic growth media and 1 mL was plated in each well of the two slides (RPMI Corning Cellgro™ RPMI, Corning cellgro Antibiotic-Antimycotic, Fisher Scientific). The dishes containing the slides were placed into the tissue culture incubator set to 5% CO_2_ (ThermoForma Series II Water-Jacketed CO_2_ incubator). UDS analysis was performed 5–11 days after plating the buffy coat, without ultraviolet light, at passaging. One chamber of each slide was irradiated with a dose of 14 J/m^2^ at 254nm, while the other chamber was covered to block the dose and serve as the internal control. The dosed slides were then incubated for 2 hours in growth medium supplemented with 10μCi/mL [^3^H]methyl thymidine to allow for incorporation of the radioactive label (Perkin Elmer). Following this incubation step was a 2 hour chase incubation in growth media supplemented with cold thymidine (Sigma), followed by fixation, drying and removing the chambers and gaskets of the slides. Forty-eight hours after the experiment, the dried slides were dipped in photographic emulsion (NTB emulsion, Carestream Health), exposed for 11 days, and then developed using Kodak fixer and developer. Once the slides had undergone photographic development, they were placed in nuclear stain which allows for quantification of silver grains within the borders of the nuclei using a 100X oil immersion objective. Four hundred nuclei were counted for each sample, by at least two independent counters. Two samples from veterans with GWI had too few surviving lymphocytes to provide quantitative analysis after this process. The subjects’ NER capacity was quantified relative to foreskin fibroblasts (FF) as the standard control for this assay and expressed as mean ± standard error %FF. The NER capacity measured for the six female controls was not significantly different from that of the three male controls (*p* = 0.65).

### RNA Isolation and RNA Sequencing

Peripheral blood mononuclear cells (PBMCs) were isolated from the subjects’ whole blood using the Isolation of PBMC protocol (Qiagen). Total RNA was purified the same day as PBMC isolation using the RNeasy total RNA isolation kit (Qiagen). The optional QIAshredder and DNase digestions steps were also performed, as outlined in the protocol. The RNAs were then snap frozen in dry ice and ethanol then placed in the −80C freezer. RNA sequencing of the samples was completed at the Nova Southeastern University Genomics Core Facility at the Center for Collaborative Research, Nova Southeastern University. Subjects’ total RNA were analyzed with using Illumina TruSeq Stranded Total RNA library preparation. Sequencing was completed on a 2 × 150 bp paired-end run using the NextSeq 500 High Output kit (300-cycle; 400 million read flow cell). The data was delivered as Fastq files by the Core; these Fastq files were then downloaded into Partek Flow data analysis software. Within Partek Flow, pre-alignment QA/QC was performed followed by the alignment of raw reads to the human reference genome assembly (hg38) using the Genomic Short-read Nucleotide Alignment Program. A post-alignment QA/QC was performed to confirm optimum alignment and reads were then quantified using the annotation model from ENSEMBL (release 88). This was conducted using the Partek E/M algorithm, which utilizes RPKM scaling for gene and transcript counts. These gene counts were then filtered, normalized, and downloaded for further downstream statistical analysis. Partek Genomics Suite statistical analysis software was used to sort the normalized gene counts for the 20 canonical NER genes. Skewing of expression of the NER pathway was analyzed with a chi-square test against a random variation in gene expression; 10 genes upregulated and 10 genes downregulated. Pairwise Student’s t-tests were performed for each gene in comparing GWI subjects’ mean to that of the controls.

**FIGURE 1:**
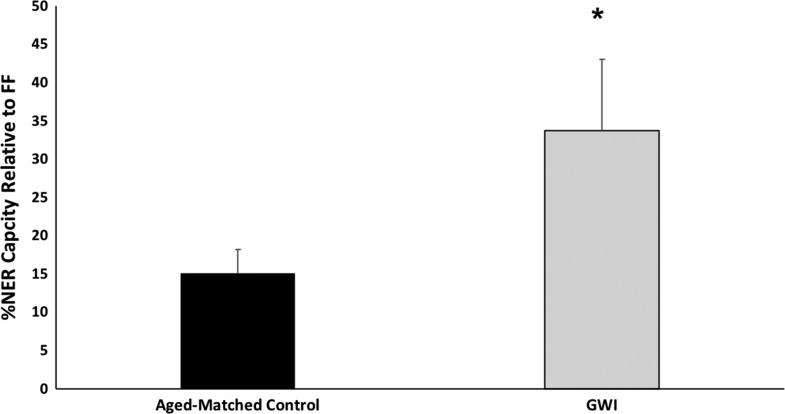
NER capacity as measured by the UDS assay of four veterans with Gulf War Illness (GWI) compared to nine age-matched controls, including one asymptomatic serviceman. NER capacities are expressed as a proportion of concurrently analyzed FF. Other controls are healthy, age-matched civilians and one asymptomatic veteran, as described in the Methods. As indicated by the asterisk, the affected population has a significantly increased mean DNA repair capacity (*p* = 0.016).

**FIGURE 2:**
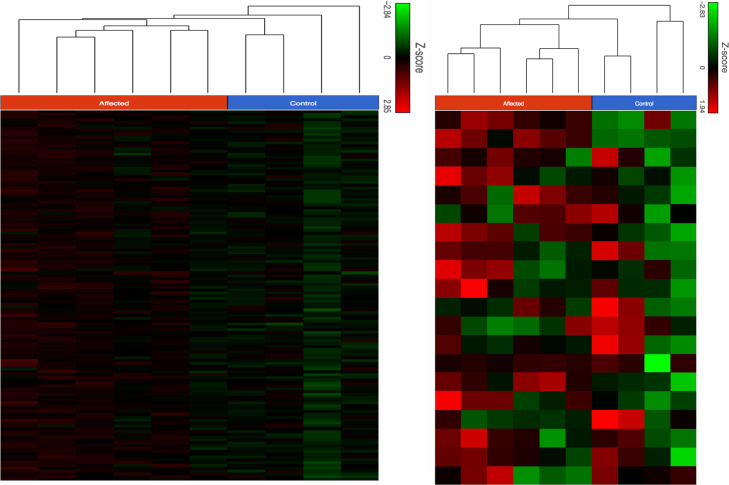
Unsupervised (left) and supervised (right) hierarchical clustering analysis of RNA sequencing data derived from lymphocytes of six veterans with GWI (Affected) and four controls, including one asymptomatic serviceman (Control). Both types of analysis distinguish the samples into two clusters, one cluster consisting of the symptomatic individuals and one cluster made up of the controls (the asymptomatic veteran is represented by the column second from left on both figures). The supervised analysis utilized gene expression data exclusively derived from the 20 canonical NER genes whose products are necessary to reconstitute activity in vitro.^21^

### Hierarchical Clustering

Unsupervised and supervised hierarchical clustering were performed using Partek Flow Software. Supervised clustering was based on the 20 canonical NER genes necessary to reconstitute activity in vitro.^21^ The Euclidean distance was determined between all data points in samples, and average linkage was used to determine distance between clusters.

## RESULTS

### NER Capacity

The lymphocyte NER capacity of four veterans with GWI and nine controls (one asymptomatic veteran and eight age-matched civilians) were determined using the UDS assay (Fig. 1). Rather than being decreased, which would have indicated a hereditary susceptibility to genotoxic exposure, the NER capacity of the GWI (15.0 ± 3.1 %FF) veterans (33.6 ± 9.3 %FF) was significantly higher than that of the control population (*p* = 0.016).

### Gene Expression Analyses

Total mRNA was extracted from lymphocytes of six GWI veterans, an asymptomatic veteran who was deployed in the Gulf War, and three local civilian controls of appropriate age. As shown in Fig. 2, left panel, unsupervised hierarchical clustering analysis, i.e., using expression of all genes, was able to distinguish the patient group from the controls. Supervised analysis, using only the 20 canonical genes in the NER pathway was equally able to separate these two groups (Fig. 2, right panel).

### NER Pathway Gene Expression Analysis

**FIGURE 3:**
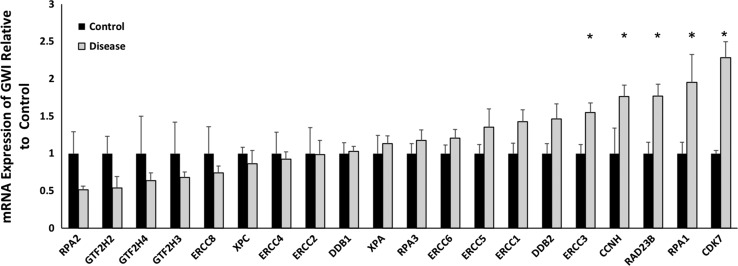
RNA sequencing analysis of affected GWI veterans (gray bars) vs. age-matched controls (black bars) for each of the 20 canonical genes of the NER pathway. All gene expression levels are normalized to the mean of the controls. Overall, 12 of the 20 genes were relatively overexpressed in the affected GWI veterans. The elevations in expression of five of the genes are individually statistically significant: *ERCC3* (*p* = 0.009), *CCNH* (*p* = 0.02), *RAD23B* (*p* = 0.005), *RPA1* (*p* = 0.04), *CDK7* (*p* = 0.0006).

We have demonstrated that gene expression of the 20 canonical genes in the NER pathway are dysregulated in association with changes in NER capacity observed during breast cancer development and progression.^22,23^ As seen in Fig. 3, the expression of the NER pathway is skewed towards overexpression in our population of affected GWI veterans, with elevated expression of twelve of twenty NER genes (*p* = 0.186). The fact that expression of five genes is significantly elevated is, in itself, significant, however (*p* < 0.001).

## DISCUSSION

These data do not seem to be consistent with our original hypothesis, that manifestation of GWI will occur preferentially in those with a hereditary susceptibility to DNA damage. Although the functional data is based on only four affected individuals and the gene expression data on six, the two analyses reinforce one another, and the NER gene expression data are able to robustly distinguish GWI veterans from unaffected and unexposed controls. However, it is difficult to imagine a scenario where hereditary resistance to DNA damage would predispose individuals to an exposure-related disease. So, is there any other possible explanation?

The UDS assay is the clinical laboratory method used to confirm a diagnosis of the NER-deficiency syndrome xeroderma pigmentosum,^24^ but it has also been widely applied in toxicology. Performed in vivo or in vitro in hepatocytes, the UDS assay identifies carcinogens through the induction of NER capacity.^25–27^ We have observed and documented epigenetic regulation of the NER pathway at the level of function and/or gene expression in tissue specificity^18,19,28^ and carcinogenesis.^22,23,29^ It is therefore possible that our results showing increased NER capacity and gene expression in GWI veterans is due to a persistent hormetic effect.

Hormesis is a process whereby exposure to a toxin induces increased resistance to its toxic effects,^30,31^ and it is known to occur in various types of cells in the blood.^32–34^ Hormesis is most accepted as a consequence of low-dose radiation exposure in the field of health physics,^35^ but there is evidence from many other fields.^31^ Indeed, since oil well fires are an accepted element in the risk profile of GWI incidence,^36^ it is reasonable to conjecture that this exposure is responsible for induction of NER, since it is necessary for the repair of so-called “bulky DNA adducts” associated with reaction with the products of organic combustion.^37^ Indeed, cigarette smoking has been shown to affect lymphocyte NER gene expression,^38^ and to confer protection against occupational exposures.^39^ The etiology of GWI is acknowledged to be complex,^40^ however, and oil fire exposure has not been shown to be the major determinant of GWI.^41^ To further complicate the interpretation of these data, there is some evidence that ionizing radiation exposure can induce expression of NER genes.^42^ Regardless of its cause, the observed hormetic increase in NER DNA repair capacity is consistent with the observations that neither bulky DNA adducts^43^ nor mutations^44^ are elevated in the DNA of lymphocytes from Gulf War veterans.

Induction of a persistent hormetic state would also be consistent with our approach to both GWI and the related disease myalgic encephalomyelitis/chronic fatigue syndrome, which we believe are manifestations of a persistently altered homeotic state.^45^ We have used both metabolomics and transcriptomics to characterize these diseases, specifically with an eye towards developing novel treatment strategies.^46,47^ NER gene expression has been found to be useful and predictive in other diseases, particularly cancer, but the pathway has usually been characterized by looking at only one^48^ or a few^49^ genes. We believe it is more powerful to use a panel of genes, since most methods of determining gene expression now provide data on the whole genome rather than single genes.^22^ We have defined the “NER score” as the average difference in expression of the 20 canonical NER genes in clinically important comparisons, and shown that it is predictive of recurrence time in childhood leukemia.^29^ This metric could certainly be applied to any veteran who is considered to have symptoms of GWI. Finally, it is becoming clear that NER expression may be modulatable in ways that could be developed into treatments.^23,49^

Further study would be necessary to determine whether the defining factor in GWI is the induction of DNA repair-associated hormesis, or the failure to return to normal repair levels in the absence of further induction. It is also not clear whether induction of a putative hormetic effect would be based exclusively on the extent and duration of exposure, or whether it would be largely determined genetically. These studies are impossible to do retrospectively, but could be done in the field. There are several blood-based methods available to directly monitor the effects of genotoxic exposure or susceptibility to such effects that could be used in concert with functional analysis of NER capacity or NER gene expression.^50^

## CONCLUSIONS

The NER pathway of DNA repair is increased in both functional capacity and gene expression in veterans with GWI. This may be indicative of an inductive effect caused by some or all of the exposures experienced in their deployment, and clinical symptoms may be associated with an inability to return to an uninduced state after removal from the causal exposures.

## PRESENTATIONS

Presented as a poster at the 2017 Military Health Systems Research Symposium.

## Notes

Opinions, interpretations, conclusions, and recommendations are those of the authors and are not necessarily endorsed by the Department of Defense.
